# Mammalian Neuronal mRNA Transport Complexes: The Few Knowns and the Many Unknowns

**DOI:** 10.3389/fnint.2021.692948

**Published:** 2021-06-15

**Authors:** Elsa C. Rodrigues, Julia Grawenhoff, Sebastian J. Baumann, Nicola Lorenzon, Sebastian P. Maurer

**Affiliations:** ^1^Center for Genomic Regulation (CRG), The Barcelona Institute of Science and Technology (BIST), Barcelona, Spain; ^2^Universitat Pompeu Fabra (UPF), Barcelona, Spain

**Keywords:** mRNA trafficking, neurons, kinesin, RNA-binding protein, mRNA localization, dynein

## Abstract

Hundreds of messenger RNAs (mRNAs) are transported into neurites to provide templates for the assembly of local protein networks. These networks enable a neuron to configure different cellular domains for specialized functions. According to current evidence, mRNAs are mostly transported in rather small packages of one to three copies, rarely containing different transcripts. This opens up fascinating logistic problems: how are hundreds of different mRNA cargoes sorted into distinct packages and how are they coupled to and released from motor proteins to produce the observed mRNA distributions? Are all mRNAs transported by the same transport machinery, or are there different adaptors or motors for different transcripts or classes of mRNAs? A variety of often indirect evidence exists for the involvement of proteins in mRNA localization, but relatively little is known about the essential activities required for the actual transport process. Here, we summarize the different types of available evidence for interactions that connect mammalian mRNAs to motor proteins to highlight at which point further research is needed to uncover critical missing links. We further argue that a combination of discovery approaches reporting direct interactions, *in vitro* reconstitution, and fast perturbations in cells is an ideal future strategy to unravel essential interactions and specific functions of proteins in mRNA transport processes.

## Introduction

Neuronal messenger RNA (mRNA) localization and local translation are crucial for a range of processes, such as neuronal development (Yoon et al., 2016; Wong et al., 2017), migration (Preitner et al., 2014), polarization (Ciolli Mattioli et al., 2019), and synaptic plasticity (Miller et al., 2002; Donlin-Asp et al., 2021). mRNAs are transported through the cytoplasm as messenger ribonucleoprotein (mRNP) complexes, with a variety of RNA-binding proteins (RBPs) bound to them to control mRNA stability, localization, and translation (Doyle and Kiebler, 2011; Buxbaum et al., 2015). Most RNA sequences important for mRNA localization are located in their 3'untranslated regions (3'UTRs). In order to establish cytoplasmic mRNA distribution, microtubule-based motor proteins, such as mostly microtubule plus-end-directed kinesins and the minus-end-directed dynein, recognize features of mRNPs that are unknown to a large extent. In this review, we focus specifically on known and potential adaptor proteins that couple mRNAs with motor proteins in mammalian neurons. This focus is motivated by the importance of revealing essential components and the architecture of the mRNA transport machinery, which will allow to disentangle the actual effect of mRNA localization from pleiotropic effects of other components of mRNA transport complexes in the future. To highlight how little is known with certainty and to emphasize potential future research goals, we discuss the value and limitations of methods often used in the past. We further provide a condensed overview on which minimal neuronal mRNA transport complexes were identified to date or can be proposed based on published evidence. We further weigh the strength of evidence for interactions that link mRNAs to motors based on the number of different methods used to show respective interactions. We differentiate explicitly between methods reporting direct and indirect interactions to highlight where further research would be beneficial. We further focus on low-throughput biochemical evidence and regard only those mRNA targets for which the binding site for the motor-coupling RBP is known. Finally, we argue that novel screening approaches detecting direct interactions between RBPs and motor proteins (Yang et al., 2018; Lang et al., 2020), advanced *in vitro* live-biochemistry assays (Heym et al., 2013; Sladewski et al., 2013; McClintock et al., 2018; Baumann et al., 2020), and fast perturbations in cells (Nishimura et al., 2009; van Bergeijk et al., 2015; Yesbolatova et al., 2020) offer a great potential to not only reveal the essential building blocks of the mammalian mRNA transport machinery, but also to discover their functionality.

## Value and Limitations of Past Approaches

**Figure 1:**
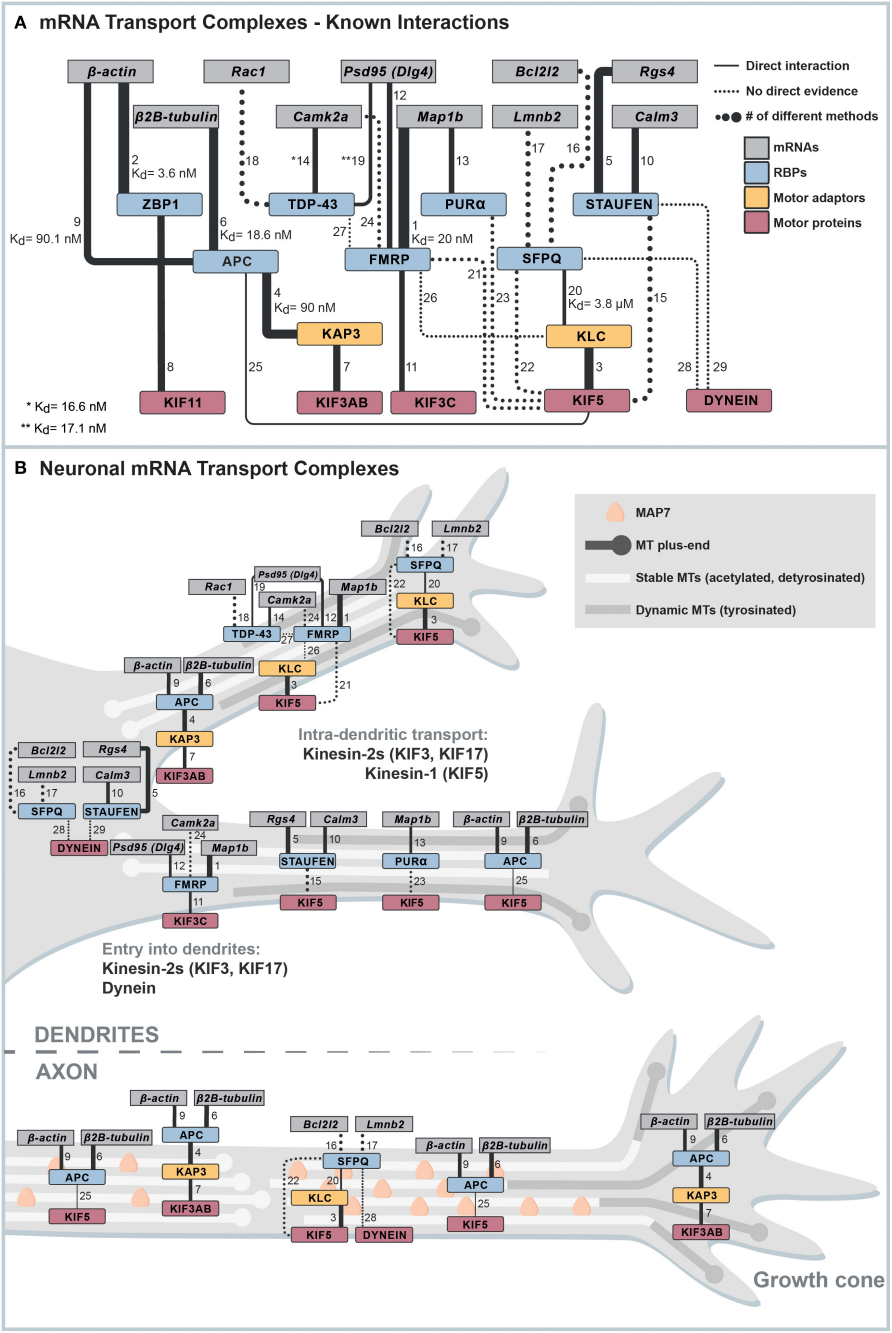
Known interactions linking mRNAs to motor proteins. **(A)** Cytoscape network of known interactions. The network summarizes and weighs available evidence that allows the establishment of connections between mRNAs and microtubule motor proteins. Only evidence from low-throughput techniques is shown, and RBP-mRNA are only plotted when the mRNA has a known binding motif for the respective RBP. The line width indicates the number of different methods used to show the respective interaction. Dotted lines indicate indirect evidence for interactions, and continuous lines are evidence for direct interactions. All evidence represented in the figure, and further evidence we excluded from the network because of different reasons is listed in Supplementary Table 1. The numbers on the lines connecting two nodes in the network correspond to the interaction number in the first column of Supplementary Table 1. **(B)** Possible minimal mRNA transport complexes for different transport steps. Combining the interactions shown in **(A)** and information about motor activity in different zones of neurites, we propose potential minimal mRNA transport complexes for different transport steps.

Important studies in the past decades have provided valuable information about which factors play a role in neuronal mRNA transport (Figure 1A, Supplementary Table 1). However, because of intrinsic limitations, many methods used cannot determine the exact functionality of a protein and its direct interactions. Results from knockdown or overexpression studies are often difficult to interpret, as it is challenging to disentangle phenotypes caused by the pleiotropic functions of proteins required for mRNA transport, such as RBPs and motor proteins. For instance, several RBPs involved in neuronal mRNA transport, such as Fragile X Mental Retardation Protein (FMRP) (Dictenberg et al., 2008; Goering et al., 2020), are also required for nuclear processes (Shah et al., 2020), nuclear mRNA export (Zhang et al., 2007; Edens et al., 2019), and translation regulation (Feng et al., 1997), whereas an mRNP-transporting motor protein like kinesin-1 KIF5 transports a variety of cargoes (Twelvetrees et al., 2010; Nakajima et al., 2012; Barry et al., 2014; Heisler et al., 2014; Ruane et al., 2016; Henrichs et al., 2020; Serra-Marques et al., 2020). Hence, analyzing the potential role of an RBP or a motor protein in mRNA transport using knockdowns or overexpressions will inevitably affect multiple cellular processes, making the interpretation of results difficult. Further, while imaging provided fascinating insights into mRNP transport dynamics and the mRNA content of neuronal mRNA transport complexes (Buxbaum et al., 2015), co-localization of an RBP and an mRNA can happen by chance unless a sufficient resolution is achieved for imaging (Eliscovich et al., 2017). Another approach, affinity purification–mass spectrometry analysis, provided valuable lists of proteins associated with neuronal mRNA transport complexes (Mallardo et al., 2003; Kanai et al., 2004; Charalambous et al., 2013; Fritzsche et al., 2013; Chu et al., 2019). However, pulldowns come with the risk of losing transiently binding dynamic interactors (Richards et al., 2021) and difficulties to distinguish between protein–protein and protein–RNA–protein interactions, in which an RNA connects two RBPs that are not directly interacting with one another. For instance, many pulled-down neuronal mRNA transport complexes are either sensitive to RNase treatment or pulldowns were done in the presence of RNase inhibitors, which leaves significant uncertainties about direct protein interactions essential for mRNA transport (Mallardo et al., 2003; Kanai et al., 2004; Dictenberg et al., 2008; Chu et al., 2019; Fukuda et al., 2021). Consequently, approaches that can reveal the core engine of mRNA transport complexes, such as biochemical reconstitution, biophysical approaches, or fast perturbations in cells, require knowledge of essential factors, direct interactions, or even minimal interaction interfaces, have been impossible for long.

## Known and Potential Messenger RNA-Motor Adaptors

Most evidence for associations with RBPs and mRNPs was reported for KIF5, a heterotetrameric kinesin comprising two heavy chains (KHCs) and two light chains (KLCs), with methods almost entirely detecting indirect interactions. FMRP was found to interact with either all KIF5 isoforms (Kanai et al., 2004), preferentially the KIF5B isoform (Zhao et al., 2020), or solely with a not specified KIF5 light chain (Dictenberg et al., 2008). Likewise, neuron-specific kinesin-2 KIF3C is a candidate for FMRP transport (Davidovic et al., 2007). While ample direct evidence exists for FMRP directly binding to localized mRNAs, such as *Map1b* (Brown et al., 2001; Menon et al., 2008) or *Psd95* (Zalfa et al., 2007), it is not clear whether FMRP can bind mRNAs and motor proteins simultaneously, which is a requirement for an mRNA-motor adaptor. Similarly, other RBPs such as PUR-alpha/beta (PURα/β) and heterogeneous nuclear ribonucleoprotein U (HNRP-U) are candidates for mRNA-motor adaptors, since they were among the strongest KIF5 tail binders in an early affinity purification study (Kanai et al., 2004). While to the knowledge of the authors there are no known links of HNRP-U and PURβ, respectively, to any localized mRNA targets, PURα is described to directly bind *Map1b* (Chen et al., 1995; Johnson et al., 2006). Furthermore, the Splicing Factor Proline and Glutamine Rich (SFPQ) immunoprecipitates in an RNase-sensitive manner with dynein and KIF5 (Fukuda et al., 2021). In addition, a fragment of SFPQ binds kinesin-1 light chain KLC1 with an affinity of 3.8 μM independent of RNA. Thus, although SFPQ is a component of RNA granules and regulates *Bcl2l2* and *Lmnb2* mRNA localization in axons (Cosker et al., 2016), it remains unclear whether KIF5-SFPQ complexes constitute the essential core of an mRNA transport complex and whether the KIF5-SFPQ interaction is sufficiently strong for processive axonal mRNA transport. Finally, another recent study has linked kinesin-1 and dynein to the transport of TDP-43-FMRP-STAU1 complexes carrying *Rac1* mRNA (Chu et al., 2019), again, using methods showing solely indirect interactions.

Another well-studied RBP required for mRNA localization is the Zipcode Binding Protein 1 (ZBP-1). Ample evidence exists for its direct binding to the beta-actin mRNA localization sequence (the “zipcode”) (Chao et al., 2010; Patel et al., 2012; Nicastro et al., 2017). Further, co-precipitation experiments show a direct interaction of the kinesin family member 11 (KIF11) with ZBP-1/Insulin Like Growth Factor 2 mRNA Binding Protein 1 (IGF2BP1) (Song et al., 2015). Nevertheless, evidence that this interaction is important for neuronal mRNA transport is lacking. Also, considering that KIF11 is not able to enter dendrites (Lipka et al., 2016), the question of how β*-actin* mRNA is transported into dendrites remains unanswered.

Another interesting candidate for mRNA-motor linkage is mRNA export factor NXF2 which binds kinesin-2 motor KIF17, KIF9, and a dynein light chain-like protein (Takano et al., 2007). Although NXF2 co-localizes with RNA granule components in hippocampal neurons, it has not been tested yet whether the NXF2-KIF17 interaction is required for neuronal mRNA transport. In addition, KIF9 has no known role in neuronal transport, and it is neither clear whether the dynein light chain-like protein functions as a part of the dynein complex, nor if dynein light chains are involved in cargo recruitment to the latter (Williams et al., 2007). Staufen2 (STAU2) is doubtlessly present in many mRNA transport complexes. It directly binds localized mRNAs (Heber et al., 2019) and affects mRNA transport (Mallardo et al., 2003; Sharangdhar et al., 2017; Bauer et al., 2019; Chu et al., 2019) but to date no direct evidence exists for its interaction with motor proteins.

Currently, the only direct evidence existing for a sufficient set of factors that can processively and selectively transport axonal mRNA packages of one to four mRNAs is based on tumor suppressor Adenomatous Polyposis Coli (APC). Using purified, full-length proteins and 3'UTR fragments, APC was shown to bind KAP3, a kinesin-2 adaptor, and G-rich motif-containing axonal mRNAs (β*2B-tubulin* and β*-actin*-mRNA fragments) with lower nanomolar affinity. Biochemical *in vitro* reconstitutions could then prove that a minimal reconstituted complex consisting of the kinesin-2 KIF3A/B/KAP3 and APC can transport RNAs over tens of micrometers (Baumann et al., 2020). While the involvement of APC in axonal mRNA localization had already been demonstrated beforehand (Preitner et al., 2014), this study underlined the value of *in vitro* reconstitution to identify essential building blocks of mRNA transport systems and their functions. As shown in Figure 1A, we summarize all known interactions that connect localized neuronal mRNAs to motors using the Cytoscape network visualization software (Cline et al., 2007). The interaction data the network is based on is listed in Supplementary Table 1. The table also contains additional data, which we excluded from the network because of different criteria specified in it.

All examples mentioned until here prove or hint at membrane-free mRNA transport complexes, but neuronal mRNPs can also associate with membrane-bound cargoes. RNAs hitchhike on slowly moving endosomes in *Xenopus laevis* retinal ganglion cells, a transport mode previously discovered in the filamentous fungus *Ustilago maydis* (Baumann et al., 2014; Pohlmann et al., 2015). In *X. laevis* ganglion cells, however, endosomes mostly serve as a translation platform, whereas most of the faster moving mRNAs move independently of endosomes (Cioni et al., 2019). In the future, it will be interesting to understand which proteins couple mRNAs to endosomes and to which extent mRNA-endosome association contributes to the actual mRNA transport process. In a different study, Annexin A11 (ANXA11), with its ordered and disordered domains, was found to function as an adaptor between liquid-like mRNPs and lysosomes (Liao et al., 2019). Even though it is intriguing to consider adaptors that link condensates to motor proteins, it is not clear currently how selectivity for certain localization signal-containing mRNAs can be inferred by such a co-partitioning-based coupling mechanism, unless mRNAs with localization signals preferentially accumulated in the same condensates that the disordered ANXA11 domain partitions into. Furthermore, to the knowledge of the authors, no data exist to date showing the co-localization of lysosomes with endogenous mRNAs. Hence, while mRNP association with endosomes and lysosomes, respectively, is an interesting phenomenon, we believe further research is required to reveal the full picture and clarify what causes mRNPs to choose endosome/lysosome association vs. direct coupling to motors *via* protein adaptors.

## Messenger Ribonuceloprotein-Transporting Motors for Different Neuronal Regions and Transport Steps

The entry of kinesins and dynein into axons and dendrites is guided by many factors, such as microtubule polarity, selectivity for microtubules with certain post-translational modifications (PTMs) (Janke and Magiera, 2020), or regulation by microtubule-associated proteins (MAPs) with specific neuronal distribution patterns (Monroy et al., 2020). Combining the information of motor-neurite selectivity and possible mRNA-motor adaptors allows postulating some mRNA transport pathways in axons or dendrites, respectively (Figure 1B).

Considering the plus-end out orientation of the microtubule cytoskeleton in axons, anterograde transport both into and inside the axon must be carried out by plus-end directed kinesin motors. Hence, the currently most likely kinesin motors with a known mRNP adaptor are kinesin-1 and kinesin-2 KIF3AB, which both bind to APC (Ruane et al., 2016; Baumann et al., 2020). Regarding dynamic tyrosinated microtubules in the growth cone, KIF3 is again the more suitable motor since MAP7, which KIF5 needs for efficient microtubule recruitment, only slowly populates freshly polymerized microtubules (Monroy et al., 2018, 2020; Hooikaas et al., 2019). Finally, if the KIF11-ZBP-1 interaction (Song et al., 2015) was to be found relevant in neurons, it could mediate axonal β*-actin* mRNA transport.

In dendrites, the minus-end out orientation of acetylated microtubules inhibits the entrance of kinesin-1 (Tas et al., 2017), whereas dynein and other kinesins can drive mRNA transport into dendrites (Lipka et al., 2016). However, to date, there is no evidence for direct interactions linking mRNAs to either dynein or any of the kinesins classified as strongly dendrite-targeting, such as KIF1A/B/C and KIF21A/B (Lipka et al., 2016). Hence, currently, the only options for postulating an mRNA transport complex for transport into dendrites with a proven direct link to mRNA include two different kinesin-2 motors, KIF3 and KIF17. Both motors can enter dendrites but apparently do so at a lower frequency compared with the above-mentioned kinesin-3 and kinesin-4 motors (Lipka et al., 2016). KIF17 is a special case, as it does not autonomously enter dendrites but instead requires dynein for transport into dendrites (Franker et al., 2016). How KIF17 is coupled to dynein, however, is not understood. While APC directly binds cargo adaptor KAP3 with a 90 nM affinity (Baumann et al., 2020), its interaction with KIF17 has been, so far, only shown with indirect methods (Jaulin and Kreitzer, 2010) and not in neurons. Hence, aside from the possibility that KIF3 also transports APC-mRNPs into dendrites, no other minimal set of factors is known to date that could transport mRNAs into dendrites.

At least for bidirectional mRNA transport along the unipolar axonal microtubule cytoskeleton, it would be important to understand how mammalian mRNPs interact with dynein. Considering that direct links between RBPs and dynein exist, as exemplified by the reconstitution of dynein-based mRNA transport using the *Drosophila melanogaster*-specific RBP Egalitarian and dynein adaptor BICD2 (McClintock et al., 2018), the existence of a mammalian linker is likely, and its identification would be a significant advancement in the mRNA transport field. A second possibility would be that inactive mRNP-carrying kinesins are transported by dynein. Several indications exist in the literature that kinesins with a demonstrated or likely function in RNP transport, such as KIF3, KIF5, and KIF17, can interact with dynein (Deacon et al., 2003; Ligon et al., 2004; Kodani et al., 2013; Franker et al., 2016; Twelvetrees et al., 2016), but further experiments are required to understand if these interactions contribute to bidirectional mRNA transport.

As recent evidence suggests that 3'UTRs often contain multiple regions that trigger localization to neurites (Mikl et al., 2021), it is possible that a single mRNA is transported by more than one motor. If this was the case, an mRNA could potentially recruit different numbers of adaptor-motor complexes based on the amount of localization motifs encoded in its 3'UTR. mRNAs with longer 3'UTRs, higher numbers of localization motifs (Tushev et al., 2018) and motors attached, would then potentially reach more distal locations, as more motors per cargo increases processivity (Furuta et al., 2013). Binding and unbinding or allosteric regulation of kinesins and dynein, respectively, could then give rise to bidirectional transport; and local cues, such as signal-induced adaptor modification or local adaptor concentrations, could fine-tune direction bias or disassembly of transport complexes, thereby producing the observed mRNA distributions.

## Perspectives

We propose that stronger emphasis on the following approaches could help to accelerate progress in the mRNA transport field:

1. Discovering essential building blocks of the neuronal mRNA transport machinery: while pulldown studies have provided a valuable pool of proteins that are involved in mRNA transport, they might fail to capture important transient interactions and do not report direct interactions. As a complementary technique, high-throughput (HT) screening for direct interactions with proper orthogonal validations could add valuable information to understand how mRNAs, mRNA-motor adaptors, and motor proteins directly interact (Yang et al., 2018; Garriga-Canut et al., 2019; Lang et al., 2020). HT screening can also help to narrow down essential linkers by interaction network analysis and function as a hypothesis-generator for subsequent mechanistic studies.
2. Acquiring quantitative data about affinities between mRNA transport complex building blocks and their mode of interaction (conventional stereospecific interactions or co-partitioning into condensates because of multivalent transient interactions): neuronal mRNA distributions are likely the result of complex dynamic processes, multiple overlapping reactions, and concentration-dependent competitive mechanisms. Revealing these processes could greatly improve the understanding of how cytoplasmic mRNA distributions are generated. As shown for a variety of cellular processes, bottom-up *in vitro* reconstitution can provide exceptional clarity and quantitative insights on dynamic processes (Loose et al., 2008; Ohya et al., 2009; Maurer et al., 2012; Sawa-makarska et al., 2020). Also, for microtubule-based mRNA transport, a number of reconstitution studies could already provide a clear picture of essential components and their function (Heym et al., 2013; Sladewski et al., 2013; McClintock et al., 2018; Baumann et al., 2020). In addition, biophysical techniques could also help to identify point mutations in individual factors that specifically impair mRNA-motor coupling. Such mutants would then again be powerful tools for functional studies on mRNA transport in cells.
3. Developing new approaches to disentangle pleiotropic functions of transport-mRNP building blocks in neuronal models: motor proteins and RBPs have multiple and sometimes redundant functions. After a knockdown, the accumulation of secondary effects and residual activities of the latter, depending on protein stability, can obscure their true functions. Hence, fast depletion and restoration of proteins, e.g., performing auxin-induced degradation (Nishimura et al., 2009; Yesbolatova et al., 2020) or optogenetics (van Bergeijk et al., 2015) in combination with endogenous mRNA transport live imaging (Turner-Bridger et al., 2018; Donlin-Asp et al., 2021) would be ideal to assess how and if a studied factor directly controls mRNA transport. To further avoid artifacts potentially caused by transient (over-)expressions (Rizzo et al., 2009; Gibson et al., 2013), such assays should be carried out using endogenously tagged proteins with, as much as possible, controlled expression levels. Finally, communicating negative results, for example at dedicated sessions during conferences, could contribute to faster progress and economical use of resources.

## Author Contributions

ECR, JG, SPM, SJB, and NL wrote the manuscript. ECR designed the figure. ECR, JG, and SPM analyzed the literature. All authors contributed to the article and approved the submitted version.

## Conflict of Interest

The authors declare that the research was conducted in the absence of any commercial or financial relationships that could be construed as a potential conflict of interest.
